# First case of B ALL with KMT2A-MAML2 rearrangement: a case report

**DOI:** 10.1186/s12885-017-3368-4

**Published:** 2017-05-23

**Authors:** Estelle Menu, Nathalie Beaufils, Fabrice Usseglio, Estelle Balducci, Marina Lafage Pochitaloff, Regis Costello, Jean Gabert

**Affiliations:** 1 Department of Biochemistry & Molecular Biology, University Hospital Nord, Marseille, France; 2 U1072 INSERM, Université de la Méditerranée, Marseille, France; 3 Genetic Department of AP-HM Marseille, Marseille, France; 4 Department of clinical onco-hematology, University Hospital of La Conception, Marseille, France

**Keywords:** Secondary ALL, *KMT2A* rearrangement, *MAML2* gene, Next-Generation Sequencing

## Abstract

**Background:**

A large number of chromosomal translocations of the human *KMT2A* gene, better known as the *MLL* gene, have so far been characterized. Genetic rearrangements involving *KMT2A* gene are frequently involved in lymphoid, myeloid and mixed lineage leukemia. One of its rare fusion partners, the *mastermind like 2* (*MAML2*) gene has been reported in four cases of myeloid neoplasms after chemotherapy so far: two acute myeloid leukemias (AML) and two myelodysplasic syndrome (MDS), and two cases of secondary T-cell acute lymphoblastic leukemia (T-ALL).

**Case presentation:**

Here we report the case of a *KMT2A - MAML2* fusion discovered by Next-Generation Sequencing (NGS) analysis in front of an inv11 (q21q23) present in a 47-year-old female previously treated for a sarcoma in 2014, who had a B acute lymphoid leukemia (B ALL).

**Conclusion:**

It is, to our knowledge, the first case of B acute lymphoblastic leukemia with this fusion gene. At the molecular level, two rearrangements were detected using RNA sequencing juxtaposing exon 7 to exon 2 and exon 9 to intron 1–2 of the *KMT2A* and *MAML2* genes respectively, and one rearrangement using Sanger sequencing juxtaposing exon 8 and exon 2.

## Background

Over 70 *KMT2A* partner genes have been reported so far in the literature. Translocation of the *KMT2A* gene, better known as the *MLL* gene, is present in 7–10% of B acute lymphoblastic leukemias, *AFF1* (or *AF4*) being the most frequent partner gene. The prognosis of B acute lymphoblastic leukemias (B ALL) with *KMT2A* gene translocations are usually poor, especially with *KMT2A-AFF1* fusions [1, 2]. One of its rare partner genes, *mastermind like 2* (*MAML2*) gene has been reported in four cases of myeloid neoplasms after chemotherapies: two cases of acute myeloid leukemia (AML) and two cases of myelodysplasic syndromes (MDS) [3]. Secondary acute lymphoid leukemia is a well-documented occurrence following topoisomerase II inhibitors and cyclophosphamide chemotherapy and/or radiotherapy. The premise that radiotherapy as well as chemotherapy are inducing t(4;11)(q21;q23) was raised before [4]. Indeed two cases of secondary T-cell acute lymphoblastic leukemia with *KMT2A-MAML2* transcripts have been described in two adolescent leukemia patients [5].

At the molecular level, much progress has been made in the identification of fusion transcripts and finding new alternatives to fluorescent in situ hybridization (FISH). In fact, the identification of fusion transcripts is possible with the Next-Generation Sequencing (NGS) technologies, such as RNA-Sequencing [6]. At the moment, this method seems to be the best for identifying new gene fusion transcripts, and is steadily improving [7]. In this case, we highlight one of the limitations of this powerful tool.

## Case presentation

### Patient’s history and clinical features

A 47-year-old female, with morbid obesity and hypertrophic cardiopathy, presented to hospital in cardiopulmonary arrest most likely due to a cardiac rhythm disturbance. During hospitalization in the cardiac section, laboratory analysis revealed anemia (Hb 105 g/L), thrombocytopenia (59 × 10^9^ platelets/L) and a total leukocyte count of 6.2 × 10^9^/L with 1% blast cells. Bone marrow aspiration showed hyper cellular marrow with agranular leukemic cells (35%). The examination of the bone marrow morphology revealed dysplasia on the three cell lineages. Flow cytometric immunophenotyping of bone marrow identified blast cells that were positive for cCD79a, CD22, CD19 and CD45, and negative for CD10. There was a history of high-grade thoracic sarcoma in the previous year, treated with surgical resection, three cycles of chemotherapy (Doxorubicin/Ifosfamide) and then radiotherapy. This patient had several thrombocytopenic episodes and encephalopathy due to ifosfamide. Hence, the patient was diagnosed with B ALL with a very poor prognosis secondary to the sarcoma treatment. Induction chemotherapy with cytarabine and anthracycline was started, but only one cycle of chemotherapy was achieved due to general deterioration, leading to an admission to intensive care. The patient deteriorated rapidly and passed away.

### Patient’s molecular features

**Fig. 1:**
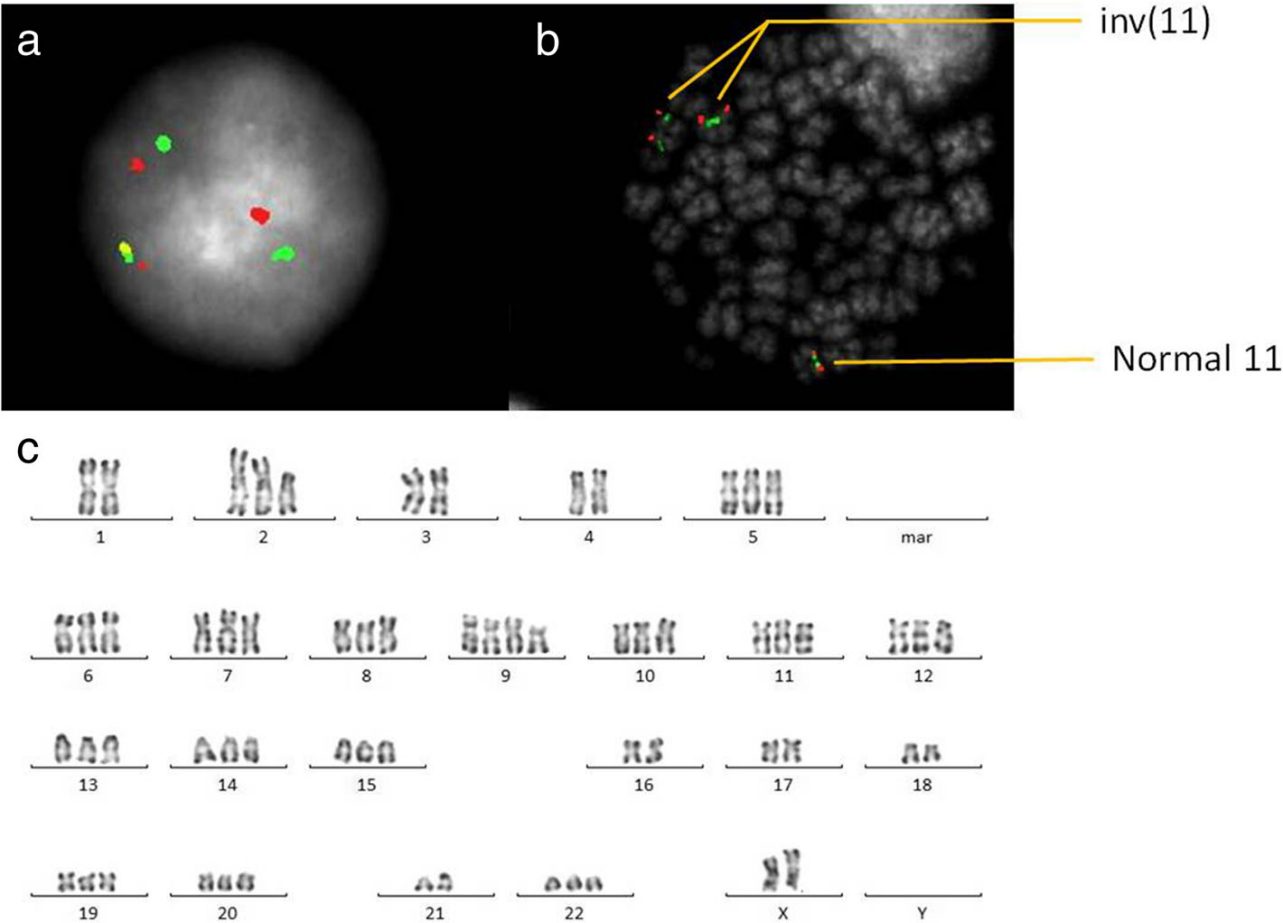
Cytogenetic analysis at initial diagnosis. Ish inv. (11) (q21, 5’MLL+)(q23, 3’MLL+) ×2 [4].nucish (Mllx3, 5’MLLsep3’MLLx2) [58/100]. FISH study using a LSI *KMT2A* break apart probe (Vysis, Abbott Molecular Inc.): (**a**) an interphase cell showing one non-rearranged orange/green signal fusion and two centomeric (red) and two telomeric (green) separate signals, (**b**) a metaphase cell showing a normal chromosome 11 and two inverted chromosome 11, (**c**) karyotype from bone marrow aspirate showing complex chromosome abnormalities

Bone marrow conventional cytogenetic analysis showed a complex hyperdiploid karyotype with multiple structural and numerical abnormalities including 2p11 deletion and duplication of the inverted chromosome 11. Based on R-banding analysis, the karyotype was interpreted as 63, XX, +del(2)(p11),+5,+6,+7,+8,+9,+9,+10,+10,+11,+12,+13,+14,+15,+19,+20,+22[22]/46, XX [4] (Fig. 1c). FISH analysis was performed on bone marrow aspirate (Fig. 1a) using a LSI *KMT2A* break apart probe (Vysis, Abbott Molecular Inc.). It revealed the presence of one normal chromosome and two inverted 11 chromosomes, each carrying a *KMT2A* gene rearrangement with an unknown fusion partner, in four metaphases and in 58/100 nuclei (Fig. 1b).

**Fig. 2:**
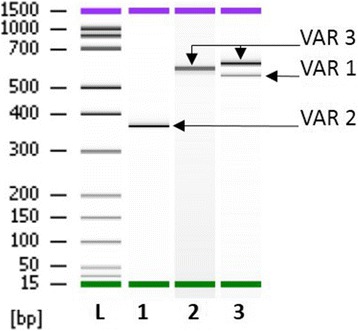
RT-PCR analysis of *KMT2A-MAML2* fusion gene. RT-PCR for the *KMT2A-MAML2* fusion was performed from patient’s RNA. 1 μg total RNA were reverse-transcribed into cDNA in a 20 μl total volume using random hexamer primers according to EAC protocol (Gabert et al.,2003). PCR reactions contained 1× PCR buffer (Applied Biosystems), 20 nmol dNTPs (Applied Biosystems), 300 nM primer of each primer, 1.25 units of AmpliTaq Gold polymerase (Applied Biosystems), and 5 μl of cDNA in a 50-μl reaction volume. Specific primers were used and were localized on exon 8 of *KMT2A* (5′-GTCCAGAGCAGAGCAAACAG-3′) and intron 1–2 of *MAML2* (5′-TCCCATCTCCAAGTCTCAGC-3′) (Lane 1), on exon 8 of *KMT2A* and exon 2 of *MAML2* (5′-GAGTCTCTCCTGGCTCCTTC-3′) (Lane 2) and on exon 7 of *KMT2A* (5′-ATCCTGCCCCAAAGAAAAGC-3′) and exon 2 of *MAML2* (Lane 3). PCR products were analyzed with Agilent DNA 1000 kit using the 2100 bioanalyzer

**Fig. 3:**
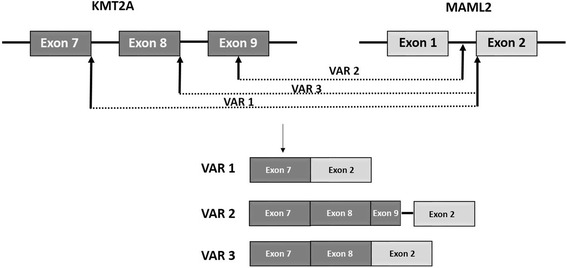
*KMT2A* and *MAML2* fusion transcripts. The *KMT2A* exon 7 and exon 8 were fused in-frame with *MAML2* exon 2 corresponding to variant 1 (VAR1) and variant 3 (VAR3) respectively. The variant 2 (VAR2) was the resulting of a breakpoint within *KMT2A* exon 9 and within *MAML2* intron 1–2

RNA sequencing (RNA-Seq) has been used to identify gene fusions on RNA extracted from the patient’s blood. Venous blood (10 mL) was collected in EDTA tubes from the patient and PBMC were isolated by erythrocytes lysis (Buffer EL, Qiagen). Total RNA was extracted from 1 × 10^7^ cells using Trizol Reagent (life technologies). For paired-end RNA-Seq, we have used the TruSeq RNA Library Prep Kits followed by paired-end cluster generation and sequencing using the 2 × 150 NextSeq 500 high output kit and the Illumina NextSeq 500 sequencing platform. The first variant (*Var 1*) corresponded to the fusion of the exon 7 of *KMT2A* and the exon 2 of *MAML2* as described previously [3]. For the second variant (*Var 2*), the data revealed that the fusion point was within the exon 9 of *KMT2A* gene and within the intron 1–2 of *MAML2* gene. To validate these RNA-Seq results, we have performed RT-PCR with specific primers localized on exon 7 or exon 8 of *KMT2A* gene and intron 1–2 or exon 2 of *MAML2* gene and Sanger sequencing (Fig. 2). The results had confirmed the presence of the two variants but had showed unexpectedly a new variant (*Var 3*). This fusion gene corresponded to the rearrangement juxtaposing exon 8 and exon 2 of the *KMT2A* and *MAML2* genes respectively and was detected with a higher expression level than the other variants (Fig. 3).

## Discussion and conclusions

Here, we reported the first case of a B ALL in a 47 years old woman bearing a *KMT2A-MAML2* rearrangement detected by NGS RNA analysis. As a result, *KMT2A-MAML2* rearrangement can be found in several types of acute leukemia. Moreover, we have detected two new variants of this fusion transcript based on exon fusion analysis. In fact, RNA sequencing juxtaposing exons 9 of the *KMT2A* to intron 1–2 of the *MAML2* gene, and exon 8 of the *KMT2A* to exon 2 of the *MAML2* gene, have not previously been reported.

Three hypotheses may explain these molecular findings. Either three different breakpoints exist, or/and it is the result of alternative splicing. Indeed, mRNA precursors could lead to the production, via alternative splicing, of a fusion transcript exon 8 of the *KMT2A* gene to exon 2 of the *MAML2* gene and exon 7 of the *KMT2A* gene to exon 2 of the *MAML2* gene. Alternative splicing are mechanisms invoked to play an important role in influencing the mutation effect [8]. The possibility of exons’ skipping or inclusion are almost unlimited, thus, any gene could potentially lead to tens or hundreds of distinctive transcripts. Moreover, we ruled out the possibility of a pre-mRNA by RNA sequencing and PCR. In fact, pre-mRNA contains both introns and exons, and requires splicing of introns to produce the final mRNA molecule containing only exons. In our case, after RNA sequencing and PCR only intron 1–2 of the *MAML2* gene persists and this variant was found in significant quantity. Each of the putative fusion transcripts was then translated into predicted amino acid sequences and each of the putative fusion proteins characterized. Two of the three variants (*Var 2* and *Var 3*) contained in-frame stops and are unlikely to encode functional proteins. For the third variant (*Var 1*), the transcript contained the boundary exons and generated putative fusion transcript.

Furthermore, this case highlights one of the limitations of NGS. In fact, two different *KMT2A-MAML2* gene fusions were found by NGS technology, but the major fusion variant, juxtaposing exons 8 of the *KMT2A* gene and exon 2 of the *MAML2* gene, was not detected. NGS methods have the ability to detect a wide array of mutation types, which is mostly limited by the need to know all potential off-target capture regions and to properly process the raw data generated by the sequencer [9]. We had to use Sanger sequencing with specific primers to detect this third variant of *KMT2A-MAML2* gene fusion, which was clearly demonstrated on the PCR gel (Fig. 2).

Concerning the development of secondary leukaemia following chemo-radiotherapy for sarcoma, it is known that chemo-radiotherapy administered for a primary cancer will increase the risk of secondary neoplasms [10]. Alkylating agents have been reported as affecting the radiation therapy-associated risk for secondary malignancies [11]. However, in this case, the speed of development of a secondary leukemia is unusual. Alkylating agent/radiotherapy-related leukemia has a mean latency period of 5–7 years, are often preceded by a myelodysplastic phase and are frequently associated with complex karyotypes, whereas acute secondary leukaemia after treatment with DNA topoisomerase II inhibitors has a relatively short latency period (several months to 3 years) and is associated with chromosomal translocation involving 11q23, respectively the *KMT2A* gene [12, 13].

To conclude, clinical outcomes of *KMT2A*-associated leukemias are often unfavorable and identification of a *KMT2A* partner could lead to new therapeutic strategies. Moreover, bringing to light several variants of a fusion transcript, such as the *KMT2A-MAML2* gene, is important in the follow-up of residual disease.

## Abbreviations

ALL: acute lymphoid leukemia
AML: acute myeloid leukemia
FISH: fluorescent in situ hybridization;
*MAML2*: mastermind like 2
MDS: myelodysplasic syndrom
NGS: Next-Generation Sequencing

## References

[1] Yokoyama A (2015). Molecular mechanisms of MLL-associated leukemia. Int J Hematol.

[2] Saleem M, Yusoff NM (2016). Fusion genes in malignant neoplastic disorders of haematopoietic system. Hematol Amst Neth.

[3] Nemoto N (2007). Identification of a novel fusion gene MLL-MAML2 in secondary acute myelogenous leukemia and myelodysplastic syndrome with inv(11)(q21q23). Genes. Chromosomes Cancer.

[4] Pagano L (1999). Acute lymphoblastic leukaemia occurring as second malignancy: report of the GIMEMA archive of adult acute leukaemia. Gruppo Italiano Malattie Ematologiche Maligne dell’Adulto. Br J Haematol.

[5] Metzler M (2008). Inv(11)(q21q23) fuses MLL to the Notch co-activator mastermind-like 2 in secondary T-cell acute lymphoblastic leukemia. Leukemia.

[6] Kumar S, Razzaq SK, Vo AD, Gautam M, Li H. Identifying fusion transcripts using next generation sequencing. Wiley Interdiscip Rev RNA. 2016. doi:10.1002/wrna.1382. 10.1002/wrna.1382 PMC5065767 27485475

[7] Scolnick JA, Dimon M, Wang I-C, Huelga SC, Amorese DA (2015). An Efficient Method for Identifying Gene Fusions by Targeted RNA Sequencing from Fresh Frozen and FFPE Samples. PLoS One.

[8] Chen M, Manley JL (2009). Mechanisms of alternative splicing regulation: insights from molecular and genomics approaches. Nat Rev Mol Cell Biol.

[9] Daber R, Sukhadia S, Morrissette JJD (2013). Understanding the limitations of next generation sequencing informatics, an approach to clinical pipeline validation using artificial data sets. Cancer Genet.

[10] Leone G, Mele L, Pulsoni A, Equitani F, Pagano L (1999). The incidence of secondary leukemias. Haematologica.

[11] Goudarzi Pour K (2016). Secondary ALL after Successful Treatment of Ewing’s Sarcoma: A Case Report. Int J Hematol-Oncol Stem Cell Res.

[12] Douet-Guilbert N (2014). MLL partner genes in secondary acute lymphoblastic leukemia: report of a new partner PRRC1 and review of the literature. Leuk Res.

[13] Rowley JD, Olney HJ (2002). International workshop on the relationship of prior therapy to balanced chromosome aberrations in therapy-related myelodysplastic syndromes and acute leukemia: overview report. Genes Chromosomes Cancer.

